# Acute Hyperglycemia May Induce Renal Tubular Injury Through Mitophagy Inhibition

**DOI:** 10.3389/fendo.2020.536213

**Published:** 2020-12-16

**Authors:** Jingyu Wang, Xiaodan Yue, Cheng Meng, Ziyan Wang, Xiaofang Jin, Xiao Cui, Juhong Yang, Chunyan Shan, Zhongai Gao, Yanhui Yang, Jing Li, Bai Chang, Baocheng Chang

**Affiliations:** ^1^ NHC Key Laboratory of Hormones and Development, Tianjin Key Laboratory of Metabolic Diseases, Chu Hsien-I Memorial Hospital & Tianjin Institute of Endocrinology, Tianjin Medical University, Tianjin, China; ^2^ Tianjin Medical University General Hospital Airport Site, Tianjin, China

**Keywords:** acute hyperglycemia, mitophagy, mitochondria, renal tubule, kidney injury

## Abstract

**Aim:**

Acute hyperglycemia is closely related to kidney injury. Oxidative stress activation and notable mitochondria damages were found under acute hyperglycemia treatment in our previous work. In the present study, we explored the dose-effect relationship and the pivotal role of mitophagy in acute hyperglycemia induced tubular injuries.

**Methods:**

Forty non-diabetic SD rats were randomly divided and treated with different concentrations of hyperglycemia respectively during the 6-h clamp experiment. Renal morphological and functional alterations were detected. Rat renal tubular epithelial cells were treated with different concentrations of glucose for 6 h. Markers and the regulation pathway of mitophagy were analyzed.

**Results:**

Significant tubular injuries but not glomeruli were observed under both light and electron microscope after acute hyperglycemia treatment, which manifested as enlargement of tubular epithelial cells, disarrangement of epithelial cell labyrinths and swelling of mitochondria. Urinary microalbumin, β2-MG, CysC, NAG, GAL, and NGAL were increased significantly with the increase of blood glucose (*P* < 0.05). ROS was activated, mitochondrial membrane potential and LC3-II/LC3-I ratio were decreased but P62 and BNIP3L/Nix were increased in hyperglycemia groups (*P* < 0.05), which were reversed by AMPK activation or mTOR inhibition.

**Conclusion:**

Acute hyperglycemia causes obvious tubular morphological and functional injuries in a dose-dependent manner. Acute hyperglycemia could inhibit mitophagy through AMPK/mTOR pathway, which would aggravate mitochondria damage and renal tubular impairment.

## Introduction

Diabetes mellitus (DM) is becoming a more threatening public health problem not only because of its high prevalence, but also owing to its high incidence and poor outcomes of vascular complications such as diabetic kidney disease (DKD). People have used to concentrate on the bad effects of chronic, longstanding hyperglycemia for a long period, but neglect the injuries resulting from acute hyperglycemia. In peoples’ traditional view, acute hyperglycemia occurred in diabetic ketosis and hypertonic hyperglycemia state could induce a series of metabolic disorders; in addition, acute hyperglycemia could suppress the insulin secretion function of β-cells transiently which is called “acute hyperglycemic toxicity”. We often encounter patients with diabetic ketosis accompanied by transient albuminuria, who are usually misdiagnosed as “diabetic kidney disease”. Does it mean rapidly elevated blood glucose could also lead to “acute hyperglycemic renal toxicity”? Patients admitted in intensive care unit (ICU) with acute hyperglycemia usually have a high prevalence of acute kidney injury (AKI), and strict control of blood glucose could obviously improve this outcome (1). In order to investigate the specific effects of acute hyperglycemia, we used hyperglycemic clamp in non-diabetic conscious rats keeping blood glucose concentration around 16.7 mmol/L for 6 h, and detected obvious morphological and reabsorption functional injuries of renal tubular epithelial cells in our previous work (2).

The abnormal increase of reactive oxygen species (ROS) in renal tubular epithelial cells is the pivotal mechanism of renal tubular injury (3, 4). In our previous study, we found acute hyperglycemia could lead to obvious renal oxidative stress activation and notable mitochondria damages including mitochondria swelling and irrecognizable mitochondrial crista (2). ROS is mainly produced by mitochondria, and excess ROS accumulation can aggravate mitochondria damages and even cell apoptosis (5). Clearing away damaged mitochondria in time is crucial for cellular homeostasis. Cells clear away damaged organelles and other components through autophagy to keep a stable state. The insufficiency of mitophagy can lead to ROS accumulation in cells which will aggravate cell injury and involve in the occurrence and development of many renal diseases. Autophagy is up-regulated and plays a protective role in drug induced acute kidney injury or acute ischemic renal injury (6–9); while it is down-regulated in both type 1 and type 2 diabetic rats (10–13). But what role might mitophagy play in acute hyperglycemia induced kidney injury is still unknown. In this study, we explored the dose-injury relationship of acute hyperglycemia induced renal tubular injury and investigated the possible role of mitophagy and its regulation pathway in acute hyperglycemia induced renal tubular injury.

## Materials and Methods

### Animals

Forty male Sprague-Dawley rats (body weight 250–280 g) supplied by Beijing HuaFuKang Bioscience Co., LTD were included in this study. All rats were maintained at 20–25°C and 50%–60% humidity on a 12-h–12-h light-dark cycle with free access to food and water. This study was permitted by the Tianjin Medical University Animal Committee, and all the animals were maintained according to the guidelines for the care and use of laboratory animals.

### Surgical Preparation

All animals received surgical placement of catheters into the left internal jugular vein which were then externalized to the back of the neck under anesthesia (10% chloral hydrate, 0.3 ml/kg body weight) after 1 week of adaptation. All the rats regained their presurgical body weight and kept in a good health condition before the clamp, which were preformed 5 days after surgery.

### Hyperglycemia Clamp Study and Sample Collection

Rats were randomly assigned to four groups, control group, hyperglycemia group A (HG-A, 11.1 mmol/L), hyperglycemia group B (HG-B, 16.7 mmol/L), and hyperglycemia group C (HG-C, 25.0 mmol/L), 10 in each group. Rats were kept in a postabsorptive state before the clamp, and stayed awake in the fixator during the clamp. A 6-h hyperglycemia-clamp procedure was performed as described in our previous study (2). 50% glucose solution was infused continuously through the catheters at the speed of 0.4–1.0, 0.6–1.2, or 1.8–2.5 ml/h, respectively, to keep blood glucose concentrations maintained around 11.1, 16.7, or 25.0 mmol/L in each hyperglycemia group after a bolus injection for about 3–5 min to raise blood glucose to the target level rapidly. Normal saline was infused in control group at the same speed as that in hyperglycemia group. Blood glucose level was determined from the tail vein every 5 min. Twenty-four–hour urine samples were collected in metabolic cages after clamp for the detection of urinary microalbumin (UMA), β2-microglobulin (β2-MG), N-acetyl-beta-D-glucosaminidase (NAG), galactosidase (GAL), neutrophil gelatinase-associated lipocalin (NGAL), and Cystatin C (CysC). Blood sample was collected from the femoral artery for serum creatinine (Scr), blood urea nitrogen (BUN) analysis, and creatinine clearance rate (Ccr) was calculated. Kidneys were isolated immediately upon the time rats were sacrificed. The left kidney was frozen for western blot and RT-PCR analysis, and the right one was kept for morphological observation.

### Morphological Observation

The kidney tissue was fixed with 4% paraformaldehyde and embedded in paraffin. Tissue sections with 4-um thickness were prepared, dewaxed in xylene, rehydrated in decreasing concentrations of ethanol and stained with hematoxylin-eosin (HE) stain. After staining, tissue sections were dehydrated in increasing concentrations of ethanol and xylene and sealed with gum. The changes of glomerular and tubular morphology were observed using light microscope (OLYMPUS IX5O/BX5O).

Kidney tissue specimens (1 mm^3^) were fixed in 2.5% glutaraldehyde and 1% osmium tetraoxide, dehydrated with gradient alcohol (50%, 70%, 90%, and 100%) and epoxypropane. Samples were then oriented longitudinally and embedded in Epon 812. Ultrathin sections were cut into 50 μm ± using ultramicrotome and then dyed with uranyl acetate and lead citrate. Transmission electron microscope (HITACHI-7500) was used to observe glomerular and tubular morphology and autophagosome.

### Renal Function Analysis

The Scr and BUN were tested using Hitachi 7600A-020 automatic biochemical analyzer, and Ccr which represented glomerular filtration function was calculated as described previously (14). Urinary UMA, β2-MG, NAG, and GAL were measured with Roche analyzer, and urinary NGAL and CysC were detected using enzyme linked immunosorbent assay (ELISA) Kits according to the manufacturers’ protocol (Wuhan Huamei Bioengineering Co., Ltd).

### Cell Culture and Treatments

Rat renal tubular epithelial cells (NRK-52E cells) purchased from Chinese Academy of Sciences Cell Library were cultured in Dulbecco’s modified Eagle’s medium (DMEM) containing 10% fetal bovine serum (Tianjin Bacchus Biotechnology Co., Ltd.) and 1% penicillin/streptomycin (Gibco) at 37°C and 5% CO_2_, and the 3–5 passages of cells were used. Cells were exposed to different concentrations of glucose (5.5, 11.1, 16.7, or 25.0 mmol/L) for 6 h. 3-(4, 5-dimethylthiazol-2-yl) -2, 5-diphenyltetrazolium bromide (MTT) assay was used to assess cell viability. NRK-52E cells were treated with hyperglycemia (16.7 mmol/L) in the presence or absence of 5’-AMP- activated protein kinase (AMPK) activator 5-Aminoimidazole-4-carboxamide1-β-D-ribofuranoside (AICAR) (500umol/L, MedChem Express) or mammalian target of rapamycin complex (mTOR) inhibitor rapamycin (50nmol/L, MedChem Express) for 6 h in order to detect the role of AMPK or mTOR in acute hyperglycemia induced kidney injury. Total RNA and protein were extracted for further analysis. Studies were replicated three times.

### ROS and Mitochondrial Membrane Potential Detection

Cells were incubated with H2-DCFDA (Cat. NO: KGAF018, KeyGEN BioTECH) for ROS detection. Media was aspirated from the cells grown in 24-well plates and the cells were washed twice with 500 μl PBS. 10μM H2-DCFDA was added to the monolayer of cells. The plate was incubated in the dark at 37°C for 30 min. After incubation, cells were washed with 500-μl PBS and then observed using fluorescence microscope (Olympus Corp., Tokyo, Japan).

Mitochondrial Membrane Potential Assay Kit (5,5’,6,6’-tetrachloro-1,1’,3,3’-tetraethylbenzimidazolcarbocyanine iodide, JC-1) (Beijing Solarbio Science & Technology Co., Ltd) was used for quantifying changes in mitochondrial membrane potential (MMP) in NRK-52E cells. JC-1 exhibits potential-driven accumulation in mitochondria, decreased red fluorescence and corresponding increased green fluorescence suggest depolarized mitochondria. Cells grown in 24-well plates were incubated with JC-1 dyeing working solution in the dark at 37°C for 20 min. After incubation, cells were washed with JC-1 dyeing buffer and examined using fluorescence microscope (Olympus Corp., Tokyo, Japan).

### Western Blotting

Renal tissues and cells were homogenized in RIPA buffer containing Protease/Phosphatase Inhibitor Cocktail (Beijing Suo Lai Bao Technology Co., Ltd.) after washing with PBS, and total protein concentration was estimated using BCA Protein Assay Kit (Wuhan Doctorate Bioengineering Co., Ltd.). Protein samples (40-80μg) were submitted to SDS-PAGE and then transferred to nitrocellulose membranes. Membranes were blocked for 2 h in 5% non-fat milk and then incubated with primary antibodies against LC3 (1:1,000, Cell Signaling), SQSTM1/p62 (1:1,000, Abcam), BNIP3L/Nix (1:1,000, Cell Signaling), mTOR (1:1,000, Cell Signaling), Phospho-mTOR (1:1,000, Cell Signaling), AMPKα (1:1,000, Cell Signaling), Phospho-AMPKα (1:1,000, Cell Signaling), SGLT2 (1:1,000, Abcam), and β-actin (1:500, Sanjian Biotechnology) at 4°C overnight. After being washed with TBST, the membranes were incubated with peroxidase-conjugated secondary antibodies (1:5000, Ai Meijie Technology Co., Ltd, China). The reactive bands were detected using the ECL system (Advansta). Signal intensity was then assessed using automatic gel imaging system (SYNGENE). Studies were replicated 3 times.

### RT-PCR Analysis

Total RNA was extracted from rat kidneys and cells using Trizol Reagent (Thermo Fisher, USA). The cDNA was synthesized using Reverse Transcription Kit (Thermo Fisher, USA) according to the following protocol: 25°C for 5min, 42°C for 60min, 70°C for 5 min. All primers were designed and synthesized by Beijing oak Biotech Corp. Primers are listed in **Table 1**. Then Quantitative real-time PCR was performed using the SYBR® Premix Ex TaqTM Kit (Dalian Bioengineering Co., Ltd.) with primers on Applied Biosystems (BIO-RAD, USA) according to the following protocol: 95°C for 1min, 94°C for 10s, 60°C for 15s, 72°C for 15s, 40 cycles. The mRNA expression levels were calculated according to the 2^−ΔΔCT^ method (15). The β-actin mRNA expression was used as reference control. Studies were replicated 3 times.

**Table 1:** Primer sequences used in the RT-PCR analysis.

Genes	Primer sequences
LC3B	Forward, 5′-CGAACAAAGAGTGGAAGATGTC-3′ Reverse, 5′-AGGCTTGGTTAGCATTGAGC-3′
P62	Forward, 5′-AGTCGGAGCGGGTTCTCTAT-3′ Reverse, 5′-GTGACACACATTCCAGCGAT-3′
BNIP3L/Nix	Forward, 5′-GCACTTCAGCAATGGGAATG-3′ Reverse, 5′-GCTCTGTTGGTATCTTGTGGTGT-3′
SGLT2	Forward, 5′-GGTCATTGCCGCGTATTTCC-3′ Reverse, 5′-ATGTTGCTGGCGAACAGAGA-3′
AMPK	Forward, 5′-TTCTGTCTGCCGTGGACTACT-3′ Reverse, 5′-CAGCCTTCCTGAGATGACCT-3′
mTOR	Forward, 5′-CCAGGAAATACCCTCTCCATC-3′ Reverse, 5′-GAAGGTCACAAAGCCGTCTT-3′

### Statistical Analysis

SPSS 20.0 was used to analyze the data. All values were tested for normality and homogeneity of variance. Normally distributed values were expressed as means ± SD. Independent t-test was used to compare differences between two groups. One-way analysis of variance was used to analyze the differences among multiple groups. Parameters that were not normally distributed were expressed as (P25, P75) and compared using Rank sum test. Values of *P* < 0.05 were considered statistically significant.

## Results

### The Establishment of Acute Hyperglycemia Model

**Figure 1:**
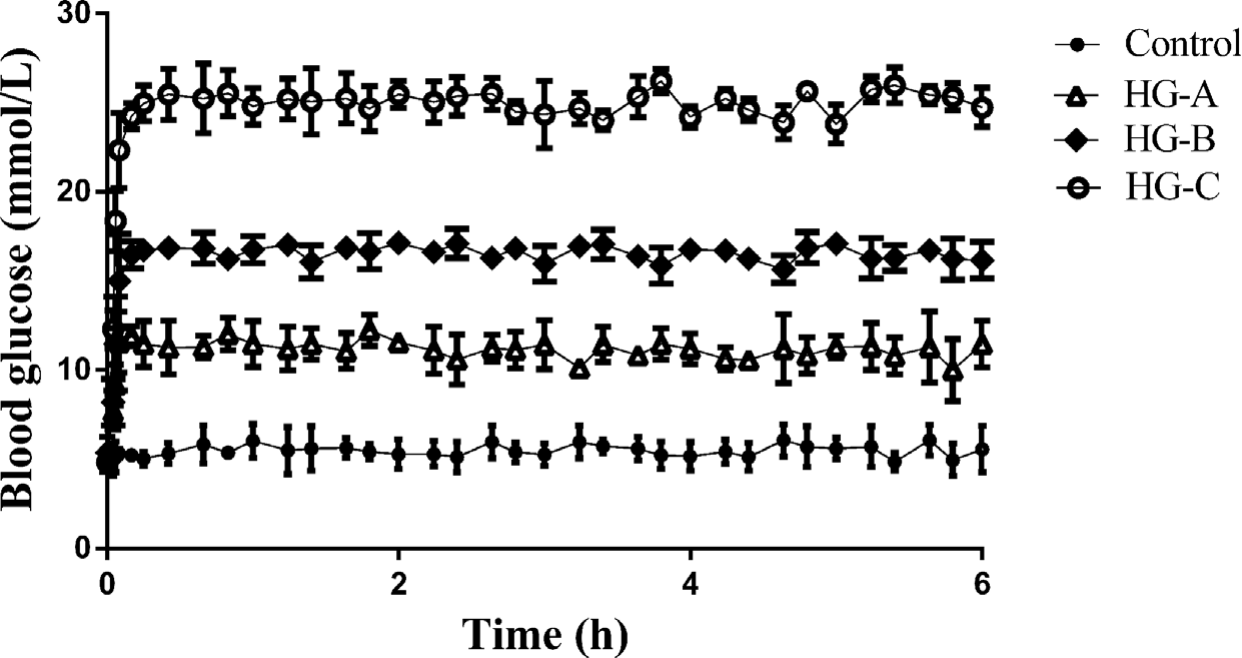
Blood glucose level during hyperglycemic clamp in different groups. N = 10 in each group. Data were expressed as means ± SD in each group. Control, control group; HG-A, hyperglycemia group A; HG-B, hyperglycemia group B; HG-C, hyperglycemia group C.

During the hyperglycemic clamp, as shown in **Figure 1**, the blood glucose level in each hyperglycemia group increased significantly compared with that in control group [11.67 ± 1.21 mmol/L, 16.67 ± 2.11 mmol/L, 24.73 ± 3.43 mmol/L vs. 5.37 ± 0.52 mmol/L, *P* < 0.05].

### Renal Morphological Alterations Under Acute Hyperglycemic State

We detected the renal glomerular and tubular morphology alterations in rats using both optical microscope and transmission electron microscope under different concentrations of acute hyperglycemia treatment.

**Figure 2:**
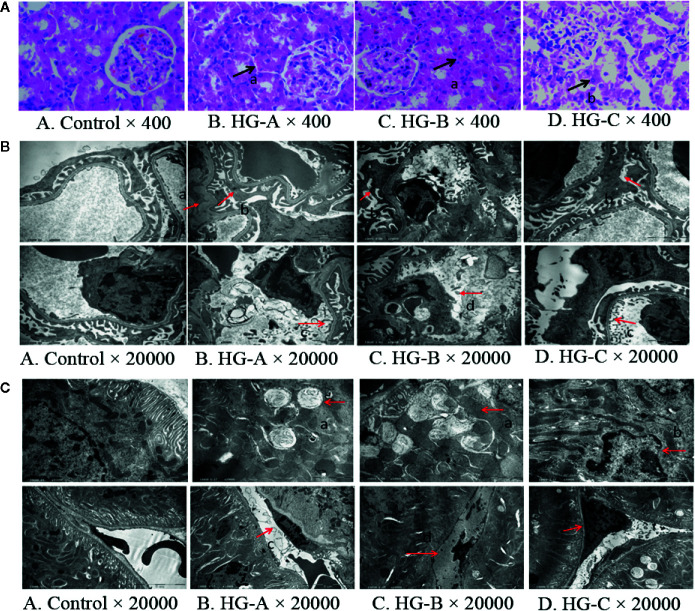
Renal morphological alterations under acute hyperglycemic state. **(A)** Renal morphological alterations under optical microscope. Arrow “a” indicates swelling of tubular epithelial cells and tubular stenosis, arrow “b” indicates fragmentation of tubular epithelial cells. **(B)** Ultrastructural alterations of glomerulus. Arrow “a” indicates thickening of glomerular basement membrane; arrow “b” indicates fusion of foot process; arrow “c” indicates enlargement of glomerular endothelial cell window; and arrow “d” indicates apoptosis of endothelial cells. **(C)** Ultrastructural alterations of renal tubular epithelial cells. Disorder of tubular epithelial cell labyrinths, vesicles development (arrow a), apoptotic changes of nucleus (arrow b) were noticed in tubular epithelial cells and swelling or apoptosis of nucleus (arrows c and d) were indicated in renal tubular vascular endothelial cells in hyperglycemia groups. Control, control group; HG-A, hyperglycemia group A; HG-B, hyperglycemia group B; HG-C, hyperglycemia group C.

Under optical microscope, swelling of tubular epithelial cells and tubular stenosis in each hyperglycemic group were noticed, which became more severe with the increase of blood glucose. However, no obvious differences in glomeruli were detected compared with that in control group (**Figure 2A**).

Under transmission electron microscope, we detected obvious foot process fusion, glomerular basement membrane thickening and endothelial cell window enlargement in acute hyperglycemia groups (**Figure 2B**). Compared to glomerular cells, more serious damages were observed in tubular epithelial cells including the disarrangement of epithelial cell labyrinths, swelling of mitochondria, irrecognizable mitochondrial crista and even apoptotic manifestation of nucleus, which also became more obvious with the increase of blood glucose concentration (**Figure 2C**).

### Alterations in Renal Function

**Figure 3:**
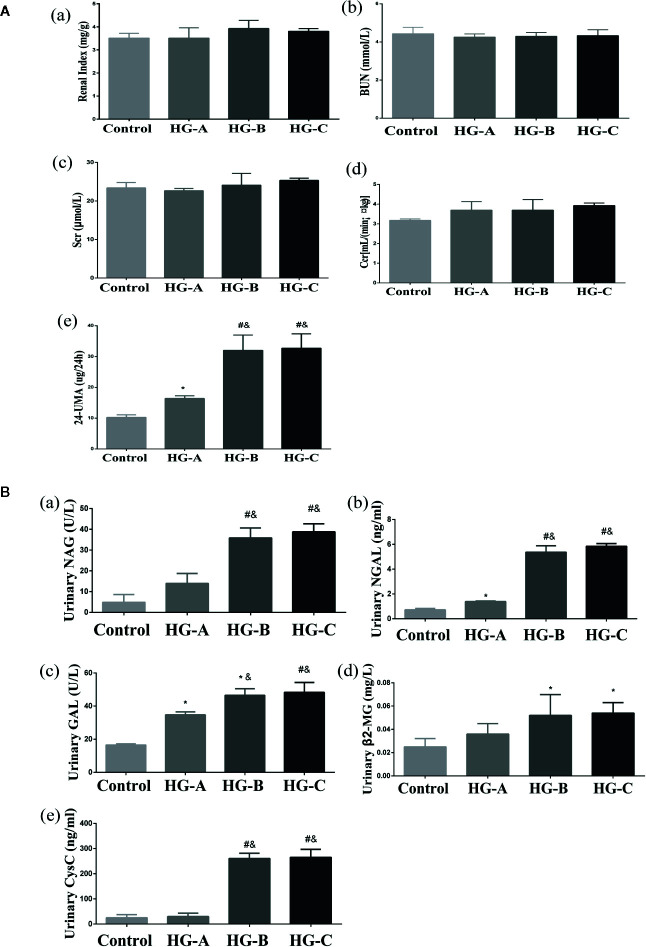
Renal glomerular function, 24-h UMA and renal tubular function alterations. N = 10 in each group. **(A)** Renal glomerular function and 24-h UMA changes. **(B)** Renal tubular function alterations. Values were expressed as means ± SD in each group. *P < 0.05 vs. Control; ^#^ *P* < 0.001 vs. Control; ^&^ *P* < 0.05 vs. HG-A. Control, control group; HG-A, hyperglycemia group A; HG-B, hyperglycemia group B; HG-C, hyperglycemia group C. BUN, blood urea nitrogen; Scr, serum creatinine; Ccr, creatinine clearance rate; 24-h UMA, 24-h urinary microalbumin; NAG, N-acetyl-β-D-glucosaminidase; NGAL, neutrophil gelatinase-associated lipocalin; GAL, β-D-galactosidase; β2-MG, β2-microglobulin; CysC, Cystatin C.

No significant differences were found for the plasma level of SCr, BUN and CCr reflecting glomerular filtration function as well as the ratio of kidney-body weight reflecting the degree of kidney hypertrophy between each two groups (*P* > 0.05). However, 24-h UMA increased significantly in hyperglycemia groups compared with that in control group, which increased gradually with blood glucose increase (16.40 ± 0.85 μg/24 h, 32.00 ± 4.95 μg/24 h, 32.70 ± 4.67 μg/24 h vs. 10.25 ± 0.84 μg/24 h, *P* < 0.05) (**Figure 3A**).

Urinary β2-MG and CysC were used to assess the reabsorption function of renal tubules, while NAG, GAL and NGAL were used to evaluate injuries of tubular epithelial cells. All these indicators increased significantly in hyperglycemia groups compared with that in control group (*P* < 0.05). As shown in **Figure 3B**, urinary GAL and NGAL began to increase significantly since blood glucose reached 11.1 mmol/L (*P* < 0.05), and urinary NAG, β2-MG and CysC began to elevate significantly since blood glucose reached 16.7 mmol/L (*P* < 0.01 for NAG, CysC and *P* < 0.05 for β2-MG vs. Control).

### Acute Hyperglycemia Could Induce Obvious Mitochondria Injuries and ROS Production

**Figure 4:**
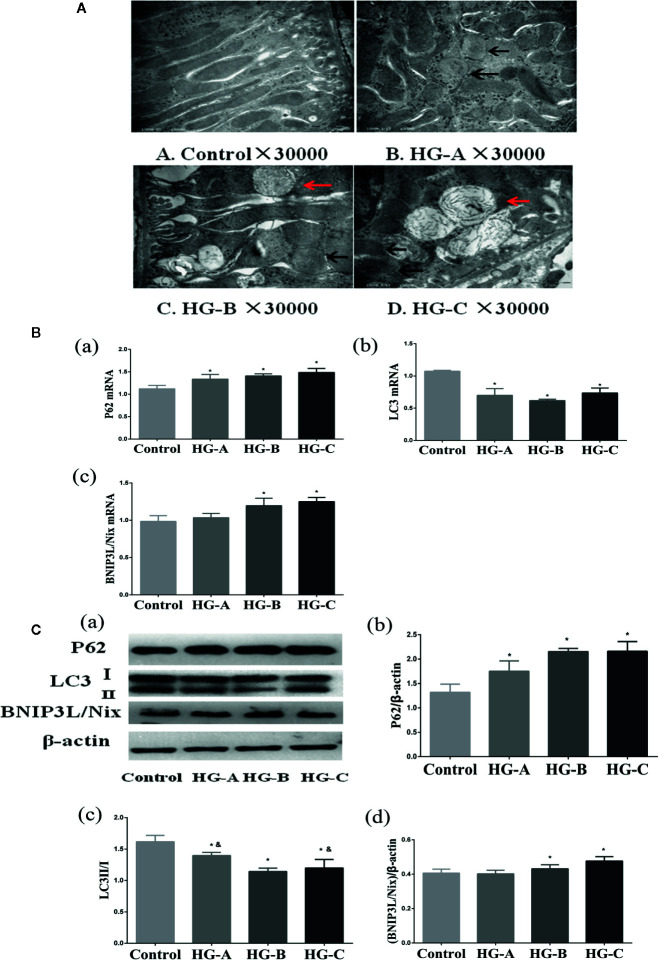
Mitophagy was inhibited in the kidney under acute hyperglycemic state. N = 10 in each group. **(A)** Swelling of mitochondria, mitochondrial fragmentation, irregular arrangement and irrecognizable mitochondrial crista observed using transmission electron microscope (black arrow). Abnormal autophagosome accumulation under transmission electron microscope (red arrow). **(B, C)** Alterations of mitophagy related markers in different groups. Values are presented as means ± SD in each group. *P < 0.05 vs. Control; ^&^ *P* < 0.05 vs. HG-B. Control, control group; HG-A, hyperglycemia group A; HG-B, hyperglycemia group B; HG-C, hyperglycemia group C.

**Figure 5:**
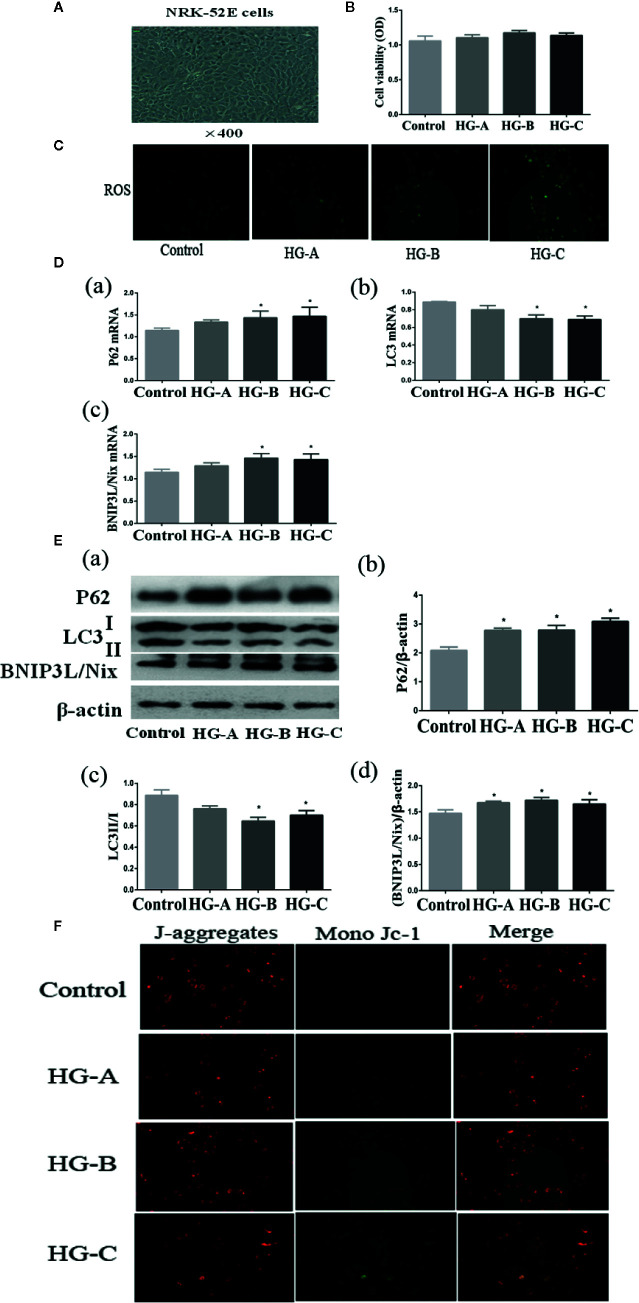
Mitophagy was inhibited in NRK-52E cells under acute hyperglycemic state. **(A, B)** Morphology and viability of NRK-52E cells. **(C)** ROS detection in NRK-52E cells (200*). **(D, E)** Alterations of mitophagy related markers in NRK-52E cells in different groups. **(F)** Mitochondrial membrane potential changes in NRK-52E cells (200*). Values are presented as means ± SD in each group. *P < 0.05 vs. Control. Control, control group; HG-A, hyperglycemia group A; HG-B, hyperglycemia group B; HG-C, hyperglycemia group C.

We observed obvious mitochondrial morphological changes after rats were treated with different concentrations of glucose, which shown as swelling of mitochondria, mitochondrial fragmentation, irregular arrangement and irrecognizable mitochondrial crista under transmission electron microscope (**Figure 4A**). We also investigated the MMP changes with JC-1 staining, and detected gradually decreased MMP with the increased glucose concentration, as evidenced by increased green fluorescence and decreased red fluorescence (**Figure 5F**). Mitochondria is the major site of ROS production. After 6-h hyperglycemia treatment, ROS accumulated gradually in tubular epithelial cells with the glucose concentration increase (**Figure 5C**).

### Acute Hyperglycemia May Induce Renal Tubular Injury Through Mitophagy Inhibition

Under transmission electron microscope, swelling of mitochondria and irrecognizable mitochondrial crista with abnormally accumulated autophagosome containing partial degraded mitochondria were detected in hyperglycemia groups, which were more serious with the increase of blood glucose concentration (**Figure 4A**). P62 reflecting the inhibition of autophagy, ratio of LC3-I to LC3-II reflecting the formation of autophagosome and BNIP3L/Nix mediating the recognition of damaged mitochondria during the process of mitophagy were studied in the present study. We detected the decrease of LC3-II/LC3-I ratio accompanied with the increase of P62 and BNIP3L/Nix in hyperglycemia groups (*P* < 0.05) (**Figures 4B, C**).

Renal tubular epithelial cells contain large amount of mitochondria, and are more sensitive to oxidative stress. From the results of both morphological and functional alterations mentioned above, we could conclude that renal tubular injuries were more severe than that of glomerulus under acute hyperglycemic state. We then treated renal tubular epithelial cells (NRK-52E cells) with different concentrations of glucose to study the effects of acute hyperglycemia on tubular epithelial cells.

As shown in **Figures 5A, B**, no obvious differences of cell viability were found in MTT assay among the four groups (*P* > 0.05). Consistent with the results *in vivo*, we also detected the gradually down-regulated LC3-II/LC3-I ratio accompanied with the gradually up-regulated P62 and BNIP3L/Nix in hyperglycemia groups (*P* < 0.05) (**Figures 5D, E**).

### SGLT2/AMPK/mTOR Pathway Played a Key Role in Acute Hyperglycemia Induced Mitophagy Inhibition in Renal Tubular Epithelial Cells

**Figure 6:**
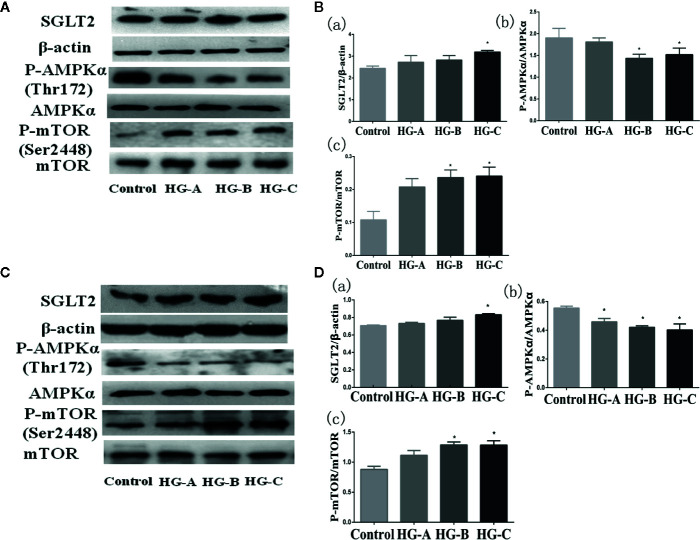
SGLT2/AMPK/mTOR pathway changes under acute hyperglycemic state both *in vivo* **(A, B)** and *in vitro* **(C, D)**. Values are expressed as means ± SD in each group. *P < 0.05 vs. Control. Control, control group; HG-A, hyperglycemia group A; HG-B, hyperglycemia group B; HG-C, hyperglycemia group C. SGLT2, sodium glucose co-transporter 2; AMPK, 5’-AMP-activated protein kinase; mTOR, mammalian target of rapamycin complex.

**Figure 7:**
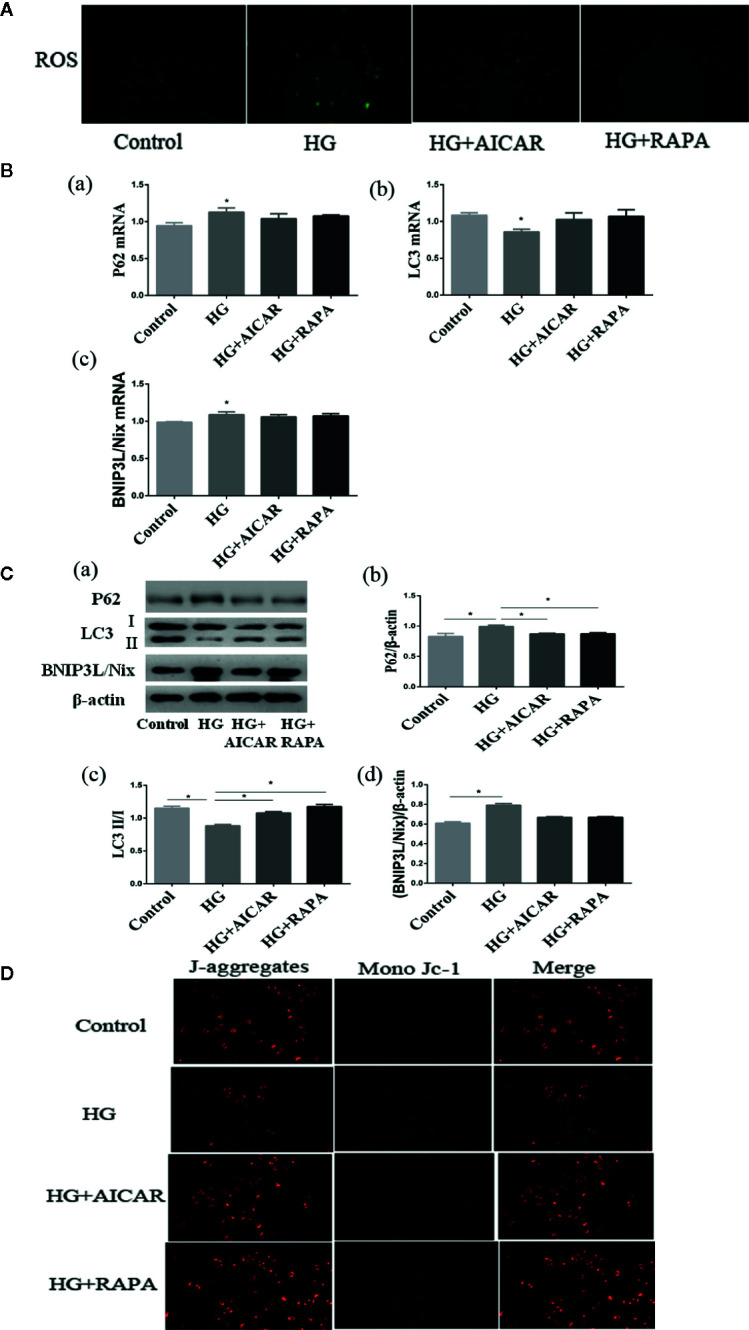
Damages could be reversed by AMPK activation or mTOR inhibition. **(A)** ROS changes in NRK-52E cells (200*). **(B, C)** AMPK/mTOR signaling pathway of mitophagy in NRK-52E cells in acute hyperglycemia group (16.7 mmol/L). **(D)** Mitochondrial membrane potential changes in NRK-52E cells (200*). Values are expressed as means ± SD in each group. *P < 0.05 vs. Control. Control, control group; HG, hyperglycemia group. AMPK, 5’-AMP-activated protein kinase; mTOR, mammalian target of rapamycin complex. AICAR, 5-Aminoimidazole-4-carboxamide1-β-D-ribofuranoside; RAPA, rapamycin.

SGLT2 mediates glucose uptake of tubular epithelial cells. The SGLT2 level was elevated after 6-h hyperglycemia treatment both *in vivo* and *in vitro* in this study (*P* < 0.05 for HG-C vs. Control). The ratio of p-AMPK/AMPK were decreased and p-mTOR/mTOR were increased by high glucose treatment (*P* < 0.05 for HG-B and C vs. Control) (**Figure 6**). In order to study the role of AMPK/mTOR in mitophagy inhibition, we further treated NRK-52E cells in hyperglycemic group (16.7 mmol/L) with AMPK activator AICAR or mTOR inhibitor rapamycin (RAPA), and detected decreased ROS production, improved MMP, increased LC3-II/LC3-I ratio and decreased P62 and BNIP3L/Nix compared with those in hyperglycemia group (*P* < 0.05) (**Figure 7**).

## Discussion

As the prevalence of diabetes is increasing rapidly, people are used to concentrate more on chronic hyperglycemia, the main clinical sign of diabetes, and its poor outcomes. But acute hyperglycemia, often occurs in diabetic patients with ketoacidosis and hyperosmolar states and non-diabetic patients with severe injuries, is usually neglected. Studies have revealed a strong relationship between acute hyperglycemia and kidney injuries. Albuminuria occurs commonly in new diagnosed diabetic patients especially those with diabetic ketosis, who are usually misdiagnosed as “diabetic kidney disease”. For critically ill patients with or without pre-diagnosed diabetes, acute elevated blood glucose could increase the incidence of acute kidney injury, and strict control of blood glucose could improve this outcome (1, 16–18). Animal studies also proved that acute hyperglycemia induced by anesthetics or serious burns could lead to glomerular filtration function impairment and tubular dilatation (19, 20). Acute hyperglycemia is usually diagnosed as random blood glucose higher than 10.0 mmol/L or 11.1 mmol/L on admission (21). In our previous study, we used hyperglycemic clamp to mimic the acute hyperglycemia state, and detected obvious glomerular and tubular injuries (2). In the present study, we further demonstrated that acute hyperglycemia could lead to renal glomerular and tubular injuries in a dose-dependent manner, and tubular injuries appeared more obvious. Hyperglycemic clamp was used in this study to increase the blood glucose of rats rapidly to a specific stable level. In this model, rats are kept fully conscious which can avoid the impacts of drugs, anesthetization or surgery on kidney. We also found that mitochondria in tubular epithelial cells were seriously injured and mitophagy was inhibited by acute hyperglycemia through AMPK/mTOR regulation.

Renal tubules manifested to be more seriously damaged under acute hyperglycemic treatment in the present study. Tubular morphological changes could be detected since blood glucose reached 11.1 mmol/L under both light and electron microscope, and became more obvious with blood glucose concentration increase. Urinary microalbumin (UMA), a traditional marker representing damages of glomerular filtration barrier and glomerular hyperfiltration, increased gradually in a dose-dependent manner since blood glucose reached 11.1 mmol/L. In recent years, UMA is believed to be primarily controlled by renal tubular epithelial cells, which represents tubular reabsorption dysfunction defined as “diabetic tubulopathy” (22–24). A good correlation was found between urinary albumin excretion and markers of tubular dysfunction. Tubular injury has been described to be a better predictor in the progression of renal disease for diabetes (25). Functions and its critical role of tubular epithelial cells are attracting more people’s eyes in recent years, and indicators of tubular injuries with higher sensitivity and specificity are springing up rapidly. Combining multiple indicators could provide more details about the type, location, severity and even the underling pathophysiological process of tubular injuries.

Plasma low molecular weight proteins (LMWP) β2-MG and CysC are freely filtered through glomerular filtration membrane and almost reabsorbed by proximal tubular epithelial cells. The raise of urinary β2-MG and CysC reflect the impaired proximal tubular reabsorption (26). In the present study, urinary β2-MG and CysC elevated significantly with blood glucose increase since blood glucose concentration reached 11.1 mmol/L, which represented acute hyperglycemia could induce obvious tubular reabsorption dysfunction in a dose-dependent manner. While urinary NAG, GAL, and NGAL are substances directly from renal tubular cells, and excreted in urine as a result of tubular epithelial cell damages. All these indicators increased significantly in hyperglycemia groups compared with those in control group (*P* < 0.05). Urinary GAL and NGAL even began to increase significantly since blood glucose reached 11.1 mmol/L (*P* < 0.05). In this study, we could conclude that acute hyperglycemia could induce obvious morphological and functional damages of tubular epithelial cells in a dose-dependent manner.

Renal tubule is responsible for the reabsorption, secretion, and excretion of many important molecules and is susceptible to various kinds of harmful factors such as ischemia, hypoxia, and oxidative stress because of its structural and functional characteristics. Proximal tubular epithelial cells are very vulnerable to high glucose damage, as they cannot decrease glucose transport to prevent excessive changes of intracellular glucose concentration when exposed to hyperglycemia (27). Blood supply of tubular epithelial cells could also be easily damaged according to its structural characteristics. Impaired microcirculation had been observed in healthy persons with acute elevated blood glucose (28, 29).

Tubular epithelial cells contain large amounts of mitochondria for their energy requirement, and an impairment of mitochondrial bioenergetics can result in renal functional decline. In our previous study, mitochondria in tubular epithelial cells were seriously damaged, and oxidative stress was obviously activated after 6-h hyperglycemia treatment (2). Oxidative stress activation in the kidney is the direct consequence of hyperglycemia, and is thought to be the core mechanism of tubular epithelial cell injuries (3, 4). In the present study, ROS increased gradually after 6-h hyperglycemia treatment on renal tubular epithelial cells, which indicated that acute hyperglycemia could activate oxidative stress in a dose-dependent manner. Intermittent glucose excursion, compared with constant hyperglycemia, was proved to activate more oxidative stress (30). Patients with isolated postprandial hyperglycemia had markedly higher urine albumin excretion, which suggested that the magnitude of glycemic spike but not the baseline glucose concentration had bad effects on urinary albumin excretion, and oxidative stress activation and free radicals generation caused by acute increase of blood glucose levels maybe the underlying mechanism (31). Mitochondria are the major source and organelle target of ROS, and mitochondrial dysfunction will trigger excessive ROS accumulation. Obvious mitochondrial morphological injuries and MMP decrease were also detected after 6-h hyperglycemia treatment. So, we concluded that acute hyperglycemia could cause serious mitochondrial damage and oxidative stress activation. Under the condition of damaged mitochondria accumulate, excess ROS increase, which would further aggravate tubular epithelial cell injuries.

Autophagy is a lysosome degradation pathway that plays an important role in maintaining intracellular homeostasis and cell integrity through removing protein aggregates and damaged or excess organelles. Autophagy was up-regulated and provided a protected effect in acute kidney injuries induced by drugs or toxins (6–9). While in both type 1 and type 2 diabetic models, autophagy was usually inhibited in tubular epithelial cells participating in the pathogenesis of DKD (11, 32). But what role may autophagy play in acute hyperglycemic kidney injuries is still not clear. LC3 is the core of autophagy. The conversion of LC3-II to LC3-I is considered as the formation of autophagosome. P62, which anchored to the autophagosome interacting with LC3, is usually degraded by autolysosome. P62 accumulates when autophagy is attenuated. In the present study, we detected the accumulated p62 and decreased expression of LC3-II/LC3-I after 6-h hyperglycemia treatment both *in vivo* and *in vitro*, which suggested the inhibited autophagy under acute hyperglycemic treatment.

Excess oxidative stress activation could aggravate mitochondria damages and even cell apoptosis, and clearing away damaged mitochondria through mitophagy in time is crucial for cellular homeostasis. Mitophagy, firstly proposed by Lemasters in 2005 (33), was defined as selectively clearance of damaged or malfunctioning mitochondria. In the kidney, mitophagy mainly occurs in proximal tubules. Mitophagy is an important component of mitochondrial quality control, which is critical for cell survival. If damaged mitochondria number exceed the clearance capacity of mitophagy, ROS accumulates, which forms a vicious circle of mitochondria injury. The observed accumulation of damaged mitochondria indicates impairment in the mitophagy system.

In the present study, we further explored the pivotal role of mitophagy and its regulatory pathway in acute hyperglycemia induced renal tubular injuries both *in vivo* and *in vitro*. Mitophagy was inhibited in a dose-dependent manner in tubular epithelial cells both *in vivo* and *in vitro* after acute hyperglycemia treatment, which was in consistence with long term hyperglycemia treatment (12, 13). The accumulated BNIP3L/Nix was detected in renal tubular cells under acute hyperglycemia treatment in this study. Nix, also known as BNIP3L, is a BH3-only proapoptotic protein. BNIP3L/Nix locates on the mitochondrial outer membrane, and was found to be important for mitochondrial elimination during erythroid cell maturation. BNIP3L/Nix was reported to depolarize mitochondria (34). CCCP induced superoxide burst could be suppressed by the deletion of Nix (35).

Kidney plays an important role in maintaining the homeostasis of blood glucose. Approximately, 180-g glucose filters through glomerulus in a healthy person every day, and is almost all reabsorbed by renal tubules, among which nearly 90% is reabsorbed by renal proximal tubules through sodium glucose co-transporter 2 (SGLT2). In the present study, SGLT2 increased gradually in hyperglycemia groups which aggravated glucose reabsorption in renal tubular epithelial cells. P62 accumulation decreased in type 1 diabetic mice after *sglt2* knocking out, which suggested SGLT2 might be involved in the regulation of autophagy activity by regulating glucose reabsorption (36).

AMPK and mTOR play a central role in the regulation of autophagy initiation. AMPK is a metabolic master-switch that regulates and maintains cellular energy homeostasis. Loss of sensitivity of AMPK activation to cellular stress impairs metabolic regulation, increases oxidative stress and apoptosis, and reduces autophagic clearance. AMPK activation will then turn off mTOR signaling and resulting in autophagy induction (37).

Activating mTOR signaling pathway could aggravate podocytes damage and glomerular filtration rate decline in patients with DKD (38). Acute glucose challenge could suppress cardiac AMPK phosphorylation and mitochondria enzyme activities in non-diabetic rats (39). Acute hyperglycemia could enhance ischemic brain damage though mTOR pathway activation, which could be relieved by mTOR inhibitor rapamycin (40). ROS production can reduce AMPK activation, then form a vicious circle that contributes to mitochondrial damage and perhaps further enhance ROS production.

Our results also showed that acute hyperglycemia could improve the phosphorylation level of mTOR, but decrease phosphorylation level of AMPK both in renal tissues of healthy rats and NRK-52E cells. Restoration through AMPK activation or mTOR inhibition could improve mitophagy activity, decrease ROS production and improve MMP in NRK-52E cells, suggesting AMPK/mTOR pathway was directly involved in the regulation of mitophagy inhibited by acute hyperglycemia induced tubular injuries.

Our study still has some limitations. First, gene knockout or overexpression model should be used to further verify the possible regulation pathway of acute hyperglycemic tubular injury; second, the reversibility of acute hyperglycemic tubular injury should be discussed in the future study.

In conclusion, acute hyperglycemia could lead to obvious tubular morphological and functional injuries when the blood glucose level was greater than 11.1 mmol/L, and behaved in a dose-dependent manner. Acute hyperglycemia could inhibit mitophagy through AMPK/mTOR pathway, which would aggravate damaged mitochondria accumulation and renal tubular injuries. The protective role of mitophagy improvement under acute hyperglycemic stress is in urgent need to be verified in the future.

## Data Availability Statement

All datasets generated for this study are included in the article/supplementary material.

## Author Contributions

JW, XY, BaiC, and BaoC conceptualized the study. XY, ZW, and XJ contributed to the methodology. XC and ZG were in charge of the data curation. JY and CS contributed to the project administration. YY and JL were in charge of the validation. JW, and BaoC acquired the funding. JW and XY wrote the original draft. BaiC and BaoC wrote, reviewed, and edited the manuscript. CM contributed to the methology and validation. All authors contributed to the article and approved the submitted version.

## Funding

This work was supported by the National Key R&D Program of China (No. 2018YFC1314000), National Natural Science Foundation of China (No. 81603461, 81774043 and 81700631), Tianjin Natural Science Foundation (No. 17JCZDJC34700, 17ZXMFSY00140 and 18ZXZNSY00280), and the fund of the State Key Laboratory of Kidney Diseases in PLA General Hospital (KF-01-133).

## Conflict of Interest

The authors declare that the research was conducted in the absence of any commercial or financial relationships that could be construed as a potential conflict of interest.
